# 

**Published:** 1977

**Authors:** 


Bristol Medico-Chirurgical Journal

(Incorporating the Medical Journal of the South-West)

Established 1883

Vol. 92 (i/ii) JANUARY/APRIL, 1977 No. 341/342

### BRISTOL MEDICO-CHIRURGICAL JOURNAL

EDITOR: D. S. REEVES, M.B., M.R.C.Path.

Published Quarterly

Annual Subscription £4 Post Free

### BRISTOL MEDICO-CHIRURGICAL SOCIETY

**President 1976/77:** Dr. S. J. Silvey.

**President-elect:** Dr. A. T. M. Roberts.

**Honorary Secretary:** A. J. Webb, Esq., 7 Percival Road, Bristol 8.

**Honorary Treasurer:** Dr. J. B. Penry, Southmead, Hospital, Bristol.

The Society was founded in 1874. In 1883 publication of the Journal began and has continued quarterly ever since then. During the years 1949 to 1962 it appeared under the title of "The Medical Journal of the South West".

### THE BRISTOL MEDICO-CHIRURGICAL JOURNAL

**Editorial Committee**

| | |
|---|---|
| Editor | Dr. D. S. Reeves, Southmead Hospital, Bristol. |
| Assistant Editor | Dr. A. P. Radford |
| Members | Dr. Rhys Davies.<br>Dr. M. B. Lennard.<br>Dr. D. W. Wright.<br>The Hon. Treasurer and Hon. Secretary of the Society. |
| Secretary | Mr. B. P. Jones, The Library, Medical School, University Walk, Bristol BS8 1TD. |

### NOTICE TO CONTRIBUTORS

The Journal is especially concerned to provide a place for the recording of medical thought, interests, and practice in and around Bristol. It therefore welcomes articles that, as well as being of immediate interest to members, will document the local and contemporary medical scene for our successors.

Original articles are invited provided that they have not been published elsewhere. References in the text should be by author's name, with year of publication in parenthesis; they should be listed at the end in alphabetical order, the name of each journal or book being given in full—not abbreviated—and the title of the article added. Illustrations should be supplied ready for reproduction. Line drawings should be in Indian ink on firm white paper or board. The author will be informed if it is found necessary to ask him to pay for the making of blocks.

The following contributions will be especially welcome; notes of forthcoming events, meetings held, degrees conferred, staff appointments, honours and public appointments and other local news.

### SURGICAL CLUB OF THE SOUTH WEST
### MEETING HELD AT THE POSTGRADUATE CENTRE, ROYAL UNITED HOSPITAL, BATH
### 22nd October, 1976
### Chairman: H. Dendy Moore

COLONOSCOPY

Dr. J. Beales, Consultant Radiologist, Royal United Hospital.

No summary available.

RESULTS OF EMERGENCY ENDOSCOPY FOR GASTROINTESTINAL BLEEDING

Dr. K. Gough, Consultant Physician, Royal United Hospital.

"An analysis of patients admitted with acute gastrointestinal bleeding to the Bath Hospitals over an 18 month period, with particular reference to the contribution made by the Endoscopy Services."

FIBEROTIC BRONCHOSCOPY

Mr. K. Jeyasingham, Consultant Thoracic Surgeon, Frenchay Hospital.

"It is now exactly one year since the Department of Thoracic Surgery at Frenchay Hospital acquired a flexible broncho-fiberoscope. Experience is now accumulating, and certain conclusions can be drawn with regard to the technique of anaesthesia, examination, the indications and the results of bronchoscopy, using this instrument. The short talk is an attempt to clarify some of these points."

THE OPTICAL URETHROTOME IN THE MANAGEMENT OF URETHRAL STRICTURE

Mr. P. Smith, Consultant Urological Surgeon, St. Martin's Hospital.

"A new instrument for the treatment of urethral stricture is described, and early impressions suggest it might well supercede all other treatments for this condition".

COMMENTS ON THE DEVELOPMENT OF THE OUT-PATIENT DIAGNOSTIC CENTRE

Dr. M. McNulty, Consultant Radiologist, Royal United Hospital.

No summary available.

TOUR OF THE DIAGNOSTIC CENTRE

LUNCH

CURRENT TREND IN THE MANAGEMENT OF MELANOMA

Mr. R. Hiles, Consultant Plastic Surgeon, Frenchay Hospital.

"An analysis of almost 1,300 cases of melanoma treated at Frenchay over the past 20 years is leading to some changes in the conventional treatment. The rationale is presented."

SCLEROSING PERITONITIS

Case presented by Mr. D. Stoney, Surgical Registrar, Royal United Hospital for Mr. T. Schofield, Consultant Surgeon, Royal United Hospital, followed by a short paper by Mr. W. Eltringham, Consultant Surgeon Bristol Royal Infirmary.

No summary available.

SOME INTERESTING EXAMPLES OF URETERIC OBSTRUCTION

Dr. M. McNulty.

No summary available.

DISCUSSION ON POSTGRADUATE SURGICAL TRAINING IN THE SOUTH WEST

1. "Training for the Fellowship", Mr. P. Smith, Consultant Urological Surgeon, St. Martin's Hospital. "Discussion on the present FRCS courses available in the South West, will be followed by some suggestions for the future".
2. "Man Power Problems", Mr. H. John, Consultant Surgeon, Royal United Hospital. "A report on the present recruiting from the Central Man Power Committee".
3. "The Role of Research in the Training Programme" Professor Johnston, Department of Surgery, University of Bristol. "Why do research? When to do research. What to research into. The MD and ChM degrees. The role of the University Department of Surgery."

4.30 - 5.00 Annual General Meeting.

### 23rd October, 1976

THE RATIONAL TREATMENT OF HAEMORRHOIDS.

Mr. Sames, Consultant Surgeon, Royal United Hospital.

"A Review of the alternative methods of treatment of haemorrhoids and their indications."

CHEMICAL LUMBAR SYMPATHECTOMY

Dr. Redman, Consultant Anaesthetist, Royal United Hospital.

"Elderly patients with severe peripheral arterio-sclerotic disease present a difficult management problem and lumbar sypmathectomy has been used as one method of treatment. This presentation will present the results of chemical lumbar sympathectomy in a series of 126 patients and show that our method of chemical lumar sympathectomy can achieve an amputation avoidance rate similar to that claimed for more extensive surgical sympathectomies".

PROBLEMS OF INTERSEX

Mr. Bamford, Consultant Gynaecologist and Obstetrician, St. Martin's Hospital.

"Review of determinants of sexual differentiation and illustration of some of the commoner forms of intersex, particularly those relevant to the general surgeon."

BOREMA REPAIRS FOR HIATUS HERNIA

Mr. John, Consultant Surgeon, Royal United Hospital.

"The paper discusses the short and long term results of the Borema operation for symptomatic hiatus hernia in the first 100 patients operated on in the Unit."

ALTERNATIVE APPROACHES TO THE KIDNEY.

Mr. Smith, Consultant Urological Surgeon, St. Martin's Hospital.

"The anterior and posterior approach to the kidney is described and compared with the more traditional approach through the loin".

### UNIVERSITY OF BRISTOL

**March 1976**

**Doctor of Medicine**

John Christopher DEAN HART

Brian David SPEIDEL

**Master of Dental Surgery**

Hubert Neil NEWMAN

**Doctor of Philosophy**

Jean ALLEN

Roger William Alfred LINDEN

Robert ORCHARDSON

Bruce MATTHEWS

**June 1976**

**Doctor of Philosophy**

Cyril Obiora ENWONWU

**Master of Surgery**

Michael DUNN

**Master of Science in Medical Sciences**

Nguyen Trung KHANH

Cosmos MARENTIS

**October 1976**

**Doctor of Medicine**

Robert Anthony BRANCH

Mansour Mabrouk Ben HALIM

Robin Harry TEAGUE

**Doctor of Philosophy**

Salah Yousif Ali AL-DAHASH

Dorothy Hanson CRAWFORD

Allen Edward GOODSHIP

Ian Roger HART

Mark James OBWOLO

Recep Selcuk YILMAZ

**January 1977**

**Doctor of Philosophy**

Gerald John ALLEN

William Keith PAVER

Martin Howard SELLENS

**Master of Science in Medical Sciences**

Beigom FAKHRAI

**March 1977**

**Master of Science in Medical Sciences**

Mohammad Razi ROUHANI-RANKOUHI

**Doctor of Philosophy**

David STANSBIE

### A SELECTION OF RECENT ADDITIONS TO THE MEDICAL LIBRARY

ANDERSON, D. J. and MATTHEWS, B.: Mastication (Wright). Presented by John Wright & Sons Ltd.

ANSELL, G.: Complications in diagnostic radiology (Blackwell).

AVERY, G. B.: Neonatology (Lippincott).

AVERY, G. S.: Drug treatment (Churchill Livingstone).

BARKER, D. J. P. and ROSE, G.: Epidemiology in medical practice (Churchill Livingstone).

BARONDES, S. H.: Neuronal recognition (Chapman & Hall).

BARTON, R.: Institutional neurosis, Ed. 3 (Wright). Presented by John Wright & Sons Ltd.

BEARD, R. W. and NATHANIELSZ, P. W.: Fetal physiology and medicine (Saunders).

BEAUGIE, J. M.: Principles of thyroid surgery (Pitman).

BECK, F. and LLOYD, J. B. eds: The cell in medical science (Acad. Pr.).

BENACERAFF, B.: Immunodeficiency and immunogenetics (M.T.P.).

BERGSMA, D.: Morphogenesis and malformation of face and brain (Liss).

BRADLEY, W. G. et al eds: Recent advances in myology: proceedings of the 3rd international conference on muscle diseases (Excerpta Medica).

BRAY, G. A.: The obese patient (Saunders).

BRUSH, M. G. and TAYLOR, R. W.: Gynaecological malignancy (Balliere).

BURROW, G. N. and FERRIS, T. F.: Medical complications of pregnancy (Saunders).

CAMPBELL, C. J. et al: Physiological optics (Harper & Row).

COLLINS, G. E. and LAZARUS, H. L.: Drug information services handbook (Publishing Sciences Group).

COMROE, J. H.: Pulmonary and respiratory physiology. 2 vols. (Dowden).

CRADDOCK, D.: A short textbook of general practice (Lewis).

CRAIN, S. M.: Neurophysiological studies in tissue culture (Raven Pr.).

CRANE, C. and WARREN, R.: Procedures in vascular surgery. Ed. 2 (Little, Brown).

CRANLEY, J. J.: Vascular surgery. Vol. 2: Peripheral venous diseases (Harper & Row).

CRIEP, L. H. Allergy and clinical immunology (Grune & Stratton).

CRYSTAL, R. G.: The biochemical basis of pulmonary function (Dekker).

DEELEY, T. J.: Principles of radiation therapy (Butterworth).

DEWHURST, C. J.: Integrated obstetrics and gynaecology for postgraduates. Ed. 2 (Blackwell).

DODD, H. & COCKETT, F. B.: Pathology and surgery of the veins of the lower limb. Ed. 2 (Churchill Livingstone).

DU PLESSIS, D. J.: Principles of surgery. Ed. 2 (Wright). Presented by John Wright & Sons Ltd.

ELDRIDGE, R. and FAHN, F. eds: Dystonia (Raven Pr.).

FAIRBANK, H. A. T.: Atlas of general affections of the skeleton. Ed. 2 (Churchill Livingstone).

FAIRLEY, G. H.: Leukaemia and lymphoma (Grune & Stratton).

FERGUSON, A. and MACSWEEN, R. N. M.: Immunological aspects of the liver and the gastrointestinal tract (M.T.P.).

FRIEDEN, E. H.: Chemical endocrinology (Acad. Pr.)

FRIES, J. F. and HOLMAN, H. R.: Systemic lupus erythematosus (Saunders).

FROHLICH, E. D.: Pathophysiology (Lippincott).

GARDNER, K. D.: Cystic diseases of the kidney (Wiley).

GARFIELD, S. L.: Clinical psychology (Arnold).

GLEN, W. W. L. et al: Thoracic and cardiovascular surgery. Ed. 3 (Appleton-Century-Crofts).

GOLD, J. J.: Gynecologic endocrinology. Ed. 2 (Harper & Row).

GREENBERGER, N. J. and WINSHIP, D. H.: Gastrointestinal disorders: a pathophysiologic approach (Year Book).

GRENFIELD, G. B.: Radiology of bone diseases. Ed. 2 (Lippincott).

GRINKER, R. R.: Neurology. Ed. 7 (Thomas).

GRUNDMANN, E. ed: Malignant bone tumors (Springer).

GRUNDMANN, E.: Glomerulonephritis (Springer).

GUTTMANN, L.: Spinal cord injuries. Ed. 2 (Blackwell).

HAMILTON, W.: Surgical treatment of endocrine disorders (Butterworth).

HANDBOOK OF PSYCHOPHARMACOLOGY, ed by L. L. Iversen et al: Section 1: Basic neuropharmacology 6 vols. (Plenum Pr.)

HARBISON, R. D.: Perinatal addiction (Spectrum).

HINCHCLIFFE, R. and HARRISON, D.: Scientific foundations of otolaryngology (Heinemann).

HOMBURGER, F.: The physiopathology of cancer. Ed. 3. 2 vols (Karger).

JAYSON, M. I. V.: The lumbar spine and back pain (Sector).

JONES, N. L. et al: Clinical exercise testing (Saunders).

JUNOD, A. and HALLER, R. D.: Lung metabolism (Acad. Pr.).

KAPLAN, H. S.: The sew sex therapy (Balliere).

KATONA, G. and BLENIGO, J. R.: Inflammation and anti-inflammatory therapy (Spectrum).

KAYE, D.: Infective endocarditis (Univ. Park Pr.).

KIMBERLIN, R. ed: Slow virus diseases of animals and man (North-Holland).

KIRKLIN, J. W.: Advances in cardiovascular surgery (Grune & Stratton).

KIRKPATRICK, C. and REYNOLDS, H.: Immunologic and infectious reactions in the lung (Dekker).

KIRSNER, J. B. & SHORTER, R. G.: Inflammatory bowel disease (Lea & Febiger).

KLAWANS, H. L.: Models of human neurological diseases (Excerpta Medica).

KNOWLES, F. G. W. and VOLLRATH, L.: Neurosecretion: the final neuroendocrine pathway (Springer).

KOSTERLITZ, H. W.: Opiates and endogenous opiod peptides (North-Holland).

KURTSIN, I. T.: Theoretical principles of psychosomatic medicine (IPST-Keter).

LANDON, D. N.: The peripheral nerve (Chapman & Hall).

LEVINE, T.: Chronic cholecystitis (IPST-Keter).

LEWIS, B.: The hyperlipidaemias: clinical and laboratory practice (Blackwell).

LIEF, H. I. and KARLEN, A.: Sex education in medicine (Spectrum).

MAGNUS, I. A.: Dermatological photobiology (Blackwell).

MAINGOT, R.: Abdominal operations. Ed. 6. 2 vols. (Butterworth).

MAKINO, S.: Human chromosomes (North-Holland).

MARKERT, C. L. and PAPACONSTANTINOU, J.: The developmental biology of reproduction (Acad. Pr.).

MARKS, R.: Common facial dermatoses (Wright). Presented by John Wright & Sons Ltd.

MARSHAK, R. H. and LINDNER, A. E.: Radiology of the small intestine. Ed. 2 (Saunders).

MASTERS, W. H. and JOHNSON, V. E.: The pleasure bond (Little, Brown).

MAXWELL, H.: Integrated medicine: the human approach (Wright). Presented by John Wright & Sons Ltd.

McALPINE, W. A.: Heart and coronary arteries: an anatomical atlas (Springer).

McLAREN, D. S. and BURMAN, D.: Textbook of paediatric nutrition (Churchill Livingstone ). Presented by Dr. D. Burman.

MENAKER, L.: Biological basis of wound healing (Harper & Row).

MOLINA, C.: Broncho-pulmonary immunopathology (Churchill Livingstone).

NAJARIAN, J. S. and DELANEY, J. P.: Surgery of the liver, pancreas and biliary tract (Stratton).

NEW, P. F. J. and SCOTT, W. R.: Computed tomography of the brain and orbit (EMI scanning) (Williams & Wilkins).

NICHOLS, P. J. R.: Rehabilitation medicine (Butterworth).

NISONOFF, A. et al: The antibody molecule (Acad. Pr.).

NYHAN, W. L.: Heritable disorders of amino acid metabolism (Wiley).

PAPPER, S. et al: Manual of medical care of the surgical patient (Little, Brown).

PARKS, M. M.: Ocular motility and strabismus (Harper & Row).

PETERS, W. and GILLES, H. M.: A colour atlas of parasitology and tropical medicine (Wolfe).

POTTER, E. L. and CRAIG, J. M.: Pathology of the fetus and the infant. Ed. 3 (Lloyd-Luke).

RANG, M.: Children's fractures (Lippincott).

ROCKWOOD, C. A. and GREEN, D. P.: Fractures. 2 vols. (Lippincott).

RORSMAN, H.: Dermatology (Wolfe).

RUSSELL, R. W. R.: Cerebral arterial disease (Churchill Livingstone).

SCHIFF, L.: Diseases of the liver. Ed. 4 (Lippincott).

SENGEL, P.: Morphogenesis of skin (Cambridge Univ. Pr.).

SHAHANI, M.: The motor system — neurophysiology and muscle mechanisms (Elsevier).

SHERMAN, M. I. and SOLTER, D.: Teratomas and differentation (Acad. Pr.)

SKYNNER, A. C. R.: One flesh, separate persons: principles of family and marital psychotherapy (Constable).

SMIDDY, F. G.: The medical management of the surgical patient (Arnold).

STOLL, B. A.: Host defence in breast cancer (Heinemann).

STRANDNESS, D. E. and SUMNER, D. S.: Hemodynamics for surgeons (Grune & Stratton).

STUART-HARRIS, C. H. and SCHILD, G. C.: Influenza — the viruses and the disease (Arnold).

SUKI, W. N. and EKNOYAN, G.: The kidney in systemic disease (Wiley).

TAVERAS, J. M. and WOOD, E. H.: Diagnostic neuroradiology. Ed. 2 (Williams & Wilkins) 2 vols.

THOMPSON, R. A. and GREEN, J. R. eds: Infectious diseases of the central nervous system (Raven Pr.)

THORBJARNARSON, B.: Surgery of the biliary tract (Saunders).

TILLEARD-COLE, R. R. and MARKS, J.: The fundamentals of psychological medicine (M.T.P.).

TOWER, D. B. ed: The nervous system. 3 vols. (Raven Pr.).

TSCHOPP, H. M.: Microsurgical neurovascular anastomoses (Springer).

VILLE, D. B. Human endocrinology: a developmental approach (Saunders).

VLODAVER, Z. et al: Coronary artery variations in the normal heart and in congenital heart disease (Acad. Pr.)

WALKER, W. F. and TAYLOR, D. E. M.: Intensive care (Churchill Livingstone).

WARREN, J. V.: Cardiovascular physiology (Dowden).

WEISS, L.: Fundamental aspects of metastasis (North-Holland).

WILKINSON, J. H.: Principles and practice of diagnostic enzymology (Arnold).

WILLIAMS, D. I. and CHISOLM, G. D.: Scientific foundations of urology. 2 vols. (Heinemann). Presented by Professor W. A. Gillespie.

YOUMANS, G. P. et al: The biologic and clinical basis of infectious diseases (Saunders).

ZATOUROFF, M.: A colour atlas of physical signs in general medicine (Wolfe).

